# Data on the derived mesoporous based catalyst for the synthesized of fatty acid methyl ester (FAME) from ternary oil blend: An optimization approach

**DOI:** 10.1016/j.dib.2020.105514

**Published:** 2020-04-22

**Authors:** T.F. Adepoju, M.A. Ibeh, U. Ekanem, A.J. Asuquo

**Affiliations:** Chemical/Petrochemical Engineering Department, Akwa-Ibom State University, Ikot Akpaden Mkpat Enin L.G.A., Akwa-Ibom State. Nigeria. P.M.B 1167, Uyo, Nigeria

**Keywords:** FAME, *Citrullus lanatus*, *Musa acuminate*, Calcination, *Cucurbita pepo*, Optimization

## Abstract

This work presents datasets on fatty acid methyl ester (FAME) synthesized from the ternary blend of Cucurbita pepo-chrysophyllum albidum -papaya mix oils via methanolysis of mesoporous CaO heterogeneous catalyst derived from the mixture of Citrullus lanatus and Musa acuminate peels. The oils were extracted from the milled powdered using the solvent extraction method. Ternary oil mixed ratio of 33:33:34 with low acid value and density was achieved using simplex lattice design software. Characterization of the mixed calcined catalyst powder (MCCP) at 700 °C for 4 h was carried out using scanning electron microscopy (SEM), energy dispersive spectroscope (EDS), X-ray diffraction analysis (XRD), and BET analysis. The thermal decomposition of mixed calcined catalyst powder (MCCP) produced 78.74% CaO with a strong basic site of 143 (μmole.g^−1^). Fatty acid methyl ester (FAME) was synthesized through the based catalyst transesterification of a derived catalyst by considering four variables data (reaction time, reaction temperature, catalyst amount and methanol/oil molar ratio) using response surface methodology (RSM). The maximum experimental FAME data of 94.29 (wt. %) was achieved at run 16, but the central composite design (CCD) software predicted value of 98.00 (wt. %) at a reaction time of 70 min, reaction temperature of 80 °C, catalyst amount of 5.0 (wt.) and methanol to oil molar ratio (MeOH/OMR) of 6.97, at the desirability of 97.90%. This was validated in triplicate, and the average FAME data obtained was 93.45 (wt. %). The produced FAME properties dataset meets the standard recommended value of ASTM and EN14214.

Specifications Table

**Table:** 

Subject	Material Science Engineering
Specific subject area	Renewable and Sustainable Energy
Type of data	Table, Figure
How data were acquired	A ternary mixture of oil was acquired through a simplex lattice mixture design. The significance of the variables was confirmed through analysis of variance (ANOVA) table. Physicochemical properties of the ternary blended oil and FAME produced were determined via AOAC (1997) standard method, Iodine value was determined through Wij's method [1].
	The developed catalyst from the mixture of calcined Citrullus lanatus and Musa acuminate peels were characterized using SEM, EDS, XRD, and BET analysis.
	Experimental design and process optimization route of converting the blended oil to FAME was achieved through the simplex lattice and central composite experimental design.
	Catalyst activities, reactor wall accumulation, and catalyst purification were performed through catalyst reusability tests.
	The produced FAME fuel properties were confirmed by comparing with [2] and [3] biodiesel recommended standard.
Data format	Raw, Analyzed
Parameters for data collection	Cucurbita pepo, Chrysophyllum albidum, and papaya seeds were milled into powders after drying. Mass extraction of oils from the powders was carried out through solvent extraction (soxhlet extractor).
	The blending of oil was carried out using simplex lattice experimental design with viscosity and acid value as the response variables.
	100 g each washed, mixed, dried and milled catalyst Citrullus lanatus and Musa acuminate peels were prepared for calcination in a furnace.
	Variable factors considered for experimental design during FAME
	production were reaction time (K1), reaction temperature (K_2_), catalyst amount (K_3_) and methanol/oil molar ratio (K_4_).
Description of data collection	Oils were extracted from the powders through the soxhlet extractor using n-hexane as solvent. Excess n-hexane in the oil was recycled using a rotatory evaporator [4].
	The ternary oil blend was achieved by experimental design using a simplex lattice mixture (Raw data); the mixture of the three oils was varied in five level-three factors design, and the response variables were the viscosity and acid value [7, 8, 9, 10, 11].
	Citrullus lanatus and Musa acuminate peels were oven-dried to constant weight at 110 °C for 3 h using an electrical oven (model DHG-9101-02). The dried samples were milled and then separated into a particle size of 0.30 mm powders [5]. The fine powders were mixed in ratio 1: 1, and then calcined at 700 °C for 4 h in a furnace
	with box-type resistance (model SX-5-12 with maximum control temperature of 1200 °C, 5 KW power rate). The calcined catalyst was then characterized by SEM, EDS, FTIR, and BET isothermal sorption (QUANTACHROME, 1 KE).
	Since the acid value (FFA <1.50) of the blended oil was within the range of successful transesterification by a based catalyst, therefore, biodiesel was synthesized through the process route earlier adopted by [6] with few modifications. Catalyst reusability was stopped after 3rd usage due to a reduction in catalyst activity.
	The physicochemical properties of the blended oil and FAME produced were determined using the standard method of AOAC. The dataset obtained were compared with the biodiesel standard.
Data source location	Department of Chemical & Petrochemical Engineering, Akwa Ibom State University, Ikot Akpaden, Mkpat Enin L.G.A., Akwa Ibom State, Nigeria.
Data accessibility	With the article

## Value of the Data

• Data on blend ratio can be used for the mixing of oils in the laboratory or industrial applications.
• Data on biodiesel synthesized can be modeled and optimized to examine the effect of variables on FAME yield
• Data will show authors in the field of engineering that calcined mixed Citrullus lanatus and Musa acuminate peels powder can produce an active CaO based catalyst for successful transesterification of oil to FAME.
• Dataset obtained shows that both calcined Citrullus lanatus and Musa acuminate peels powder can be used as a catalyst for FAME synthesized, but it mixed produced higher CaO conversion.
• Data on the physicochemical properties of the mixed oil and FAME produced shows that the produced FAME can serve as an alternative to conventional diesel.

## 1 Data

**Fig. 1:**
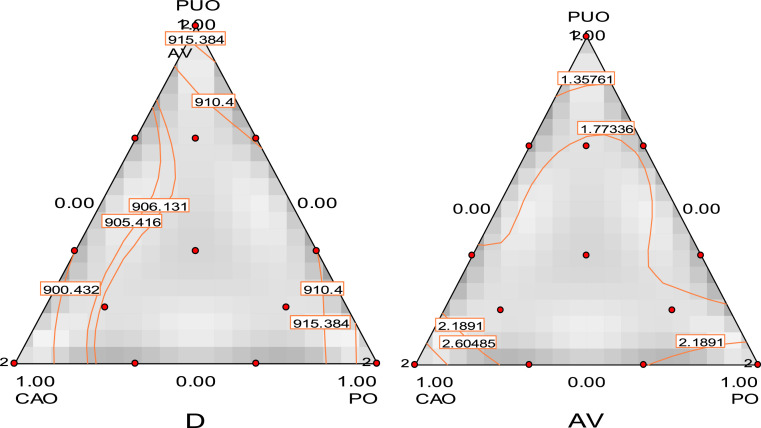
Plots of ternary model blend of oils.

**Fig. 2:**
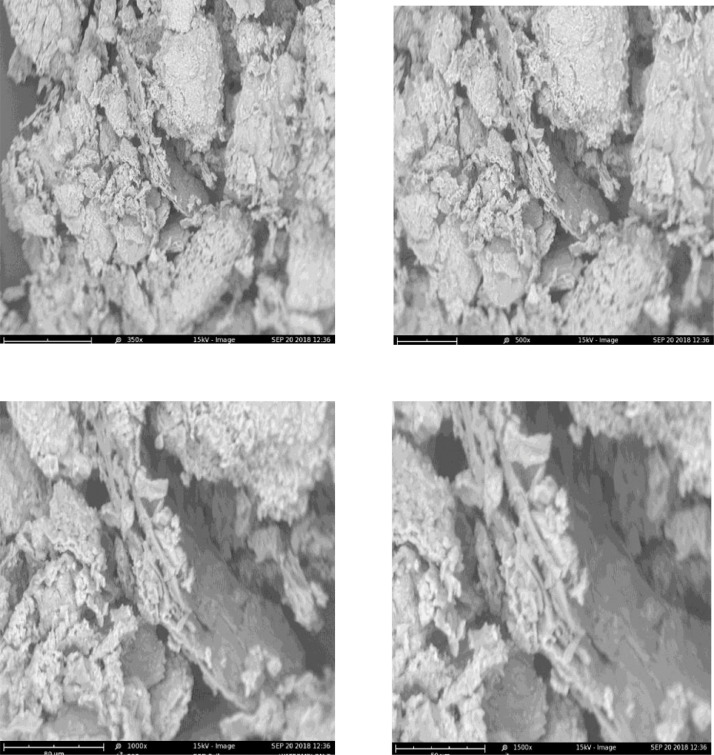

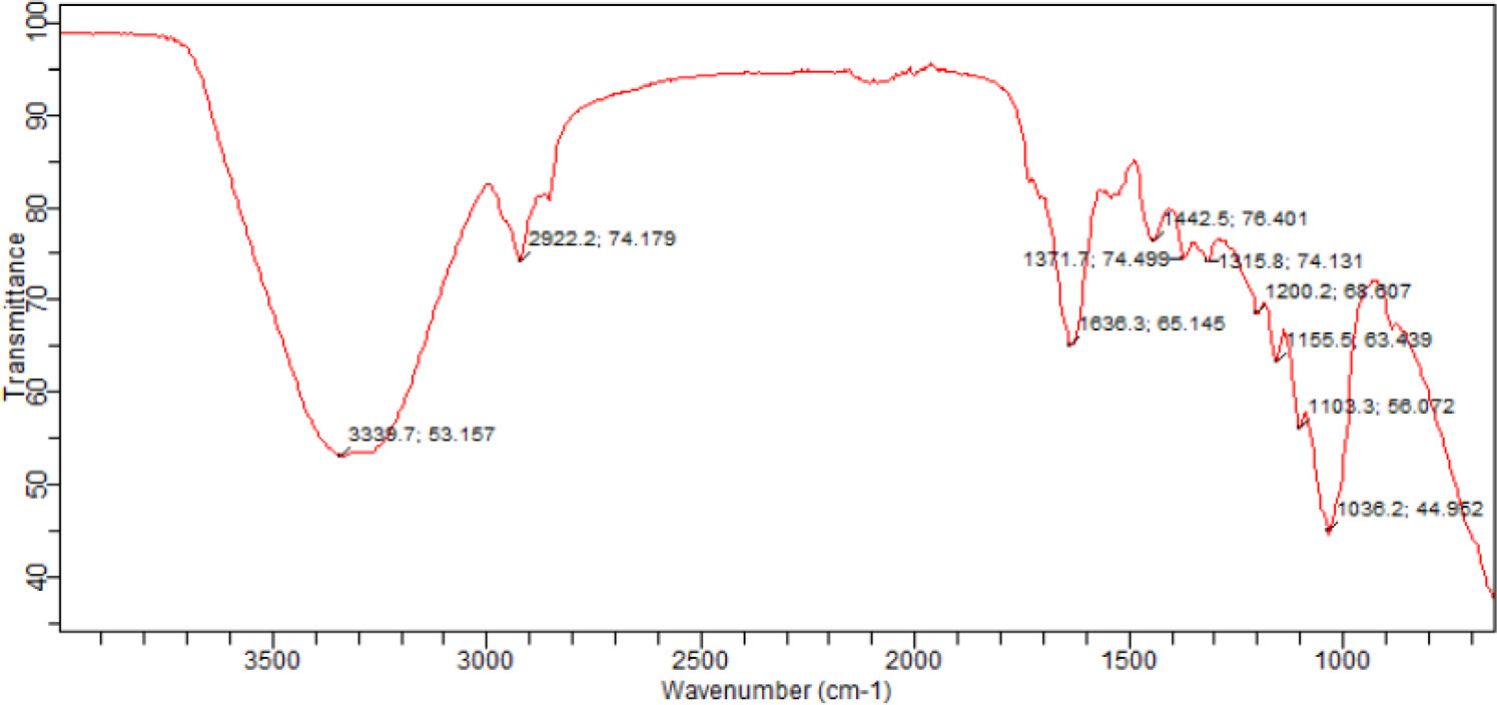
(a) SEM image of a catalyst at different magnification. (b) FTIR analysis of the catalyst.

**Fig. 3:**
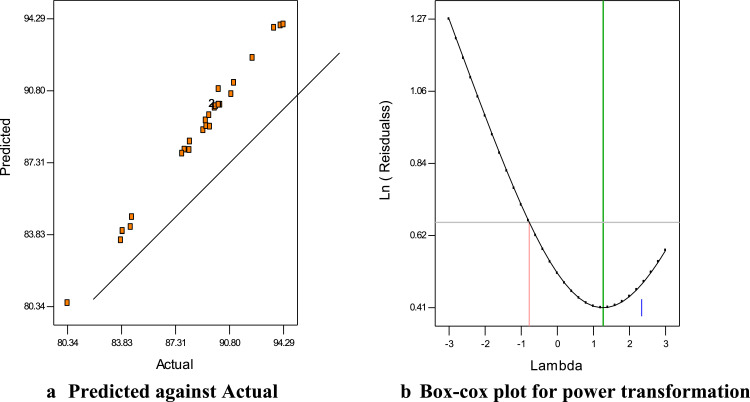
(a) Predicted against Actual. (b) Box-cox plot for power transformation.

**Fig. 4:**
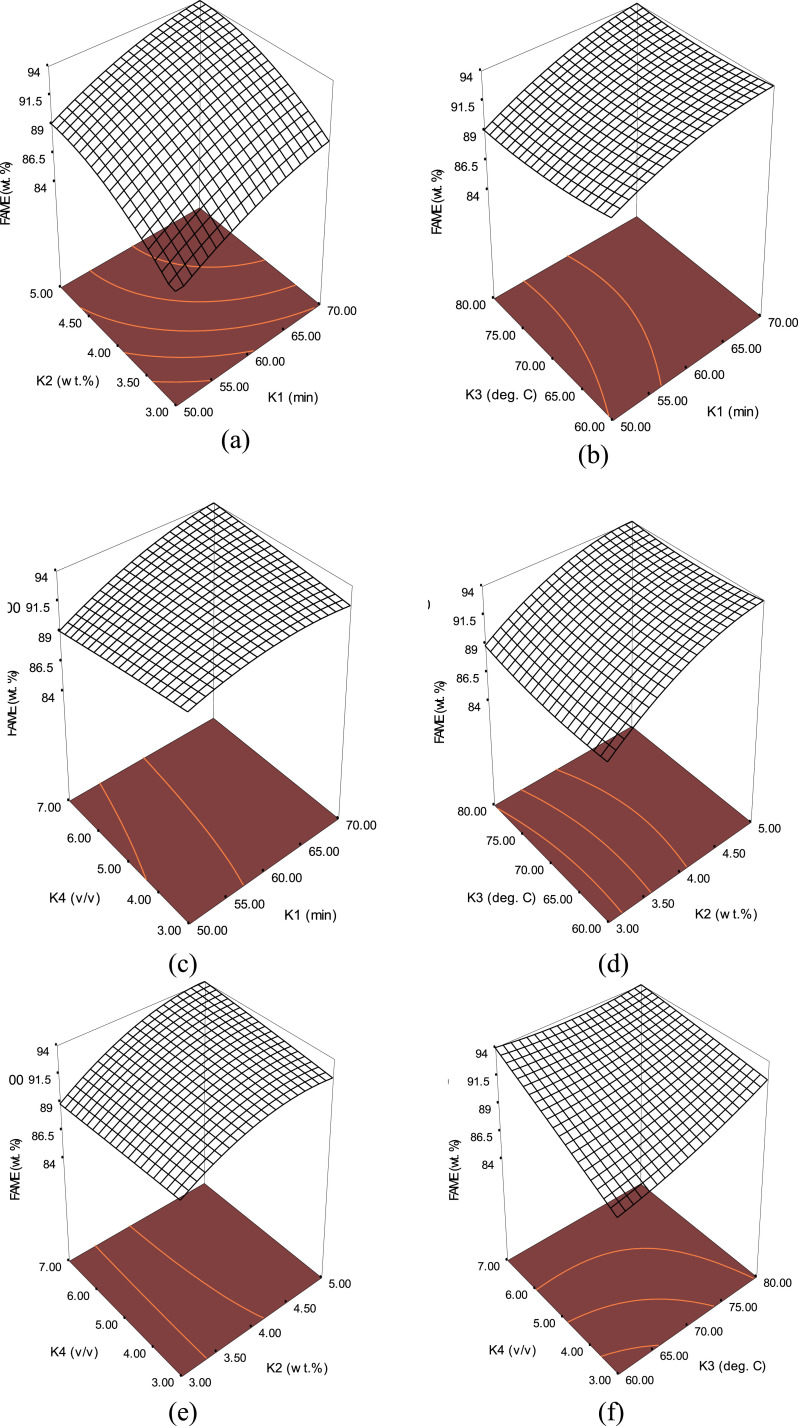
(a-f): 3-D's plots.

The dataset in this article describes the oil blend ratio which was carried out through simplex lattice design (expert 6.0.8 trial version) with three-factors (oils)-five levels design. Table 1a and1b) shows the factors, the level and the results of the 16 experimental runs with response variables’ (density and acid value), these values were used in the laboratory to obtain the experimental biodiesel yield. Table 2a and 2b shows the data on the ANOVA for a mixture of a cubic model as well as the point prediction, this was obtained through statistical analysis by a simplex lattice. Eqs. (1) and (2) showed the final equation in terms of real component generated by a design expert to show the density (d) and the acid value (AV) relationship with the variables data. Fig. 1(a-b) describes the ternary model blend ratio generated through the optimization technique of the design expert. Fig. 2(a) describes the results of the SEM used to determine the morphological characteristic of the derived catalyst, while Fig. 2b shows the FTIR used to confirm the presence of functional groups and verify the presence of characteristic absorption bands of CaO (Table 1c). Table 3 shows the data obtained for the BET surface, basicity, total pore volume, and the percentage composition of CaO obtained by EDX-nitrogen adsorption-CO2 TPD from the calcined catalysts (Citrullus lanatus, Musa acuminate peels, and the mixed). The experimental design factor, the coded level, the experimental, the predicted and the residual data are presented in Table 4a. These datasets are used for experimental modeling and statistical analysis through CCD. Table 4b describes the results of the tests of a significant and fit statistic obtained through statistical optimization, while the final equation in terms of the coded value based on a dataset that relates the response FAME with the variable data generated through the polynomial quadratic model are presented in Eq. (3). The results of the relationship between the predicted and experimental yield as well as the Box-cox plot for power transformation are presented in Fig. 3(a-b). These plots were used to know the difference between the real experimental value and the predicted value by the design expert. Fig. 4 (a-f) shows the three-dimensional interactive effect of data variables (P_1_P_2_; P_1_P_3_; P_1_P_4;_ P_2_P_3_; P_2_P_4_ or P_3_P_4_) on the output (FAME), the plots explained the relationship that exists between the interaction of the variable factors on the response of FAME. Table 5 describes the qualities of the FAME and the blended oil obtained from a ternary mix of Cucurbita pepo oil (CPO), Chrysophyllum albidum oil (CAO) and Papaya oil (PO).

(1) $$\begin{array}{cc} D= & \;918.43X_{1}+890.46X_{2}+920.37X_{3}-11.28X_{1}X_{2}-39.97X_{1}X_{3}+8.63X_{3}X_{2}+56.69X_{1}X_{3}X_{2}\\ & \;-44.48X_{1}X_{2}\left(X_{1}-X_{2}\right)+7.28X_{1}X_{3}\left(X_{1}-X_{3}\right)+78.76X_{3}X_{2}\left(X_{2}-X_{3}\right)\; \end{array}$$

(2) $$\begin{array}{cc} AV= & \;0.53X_{1}+3.02+2.61X_{3}-0.25X_{1}X_{2}\mp 0.44X_{1}X_{3}-3.11X_{3}X_{2}+2.23X_{1}X_{3}X_{2}\\ & \;+4.44X_{1}X_{2}\left(X_{1}-X_{2}\right)+5.44X_{1}X_{3}\left(X_{1}-X_{3}\right)-1.64X_{3}X_{2}\left(X_{2}-X_{3}\right) \end{array}$$

**Table 1a:** Five level three factors experimental design for oil blend.

Variable	Units	Symbol	Levels				
			-2	-1	0	1	2
*CPO*	(ml)	*X*_1_	0	0.16667	0.3333	0.66667	1.0000
CAO	(ml)	*X*_2_	0	0.16667	0.3333	0.66667	1.0000
PO	(ml)	*X*_3_	0	0.16667	0.3333	0.66667	1.0000

**Table 1b:** Experimental runs with the response variables.

R	*CPO*	CAO	PO	*CPO*	CAO	PO	D (kg/m^3^)	AV (mg KOH/g oil)
2	1.0000	0.0000	0.00	100.0000	0.0000	0.0000	918.00	0.53
9	0.6667	0.3333	0.00	66.6667	33.3333	0.0000	903.00	1.63
13	0.6667	0.0000	0.3333	66.6667	0.0000	33.3333	911.00	1.72
16	0.3333	0.6667	0.0000	33.3333	66.6667	0.0000	901.00	1.80
**15**	**0.3333**	**0.3333**	**0.3333**	**33.3333**	**33.3333**	**33.3333**	**907.00**	**1.81**
12	0.3333	0.0000	0.6667	33.3333	0.0000	66.6667	910.00	1.61
3	0.0000	1.0000	0.0000	0.0000	100.0000	0.0000	890.00	3.02
10	0.0000	0.6667	0.3333	0.0000	66.6667	33.3333	908.00	2.07
6	0.0000	0.3333	0.6667	0.0000	33.3333	66.6667	906.00	2.18
14	0.0000	0.0000	1.0000	0.0000	0.0000	100.0000	920.00	2.61
8	0.6667	0.1700	0.1667	66.6667	16.6667	16.6667	907.00	1.82
5	0.1667	0.6667	0.1667	16.6667	66.6667	16.6667	907.00	1.88
4	0.1667	0.1667	0.6667	16.6667	16.6667	66.6667	908.00	1.86
7	1.0000	0.0000	0.0000	100.0000	0.0000	0.0000	918.00	0.53
1	0.0000	1.0000	0.0000	0.0000	100.0000	0.0000	890.00	3.02
11	0.0000	0.0000	1.0000	0.0000	0.0000	100.0000	920.00	2.61

R= runs, V = viscosity, D = density and AV = acid value

**Table 1c:** Peak assignment in the spectrum.

Wavelength (cm^−1^)	1036.2 to 1442.5	1555.5 to 1636.3	2922.6 to 3338.7
Transmittance0	44.962 to 76.401	63.439 to 66.145	74.179 to 53.157
Functional group	Bending vibration of	C-H for sp^3^ carbon,	Presence of O-H of carboxylic acid and C-H
	O-Ca-O group.	C=O for sp^2^ carbon	
	Presence of sp^2^ in aldehyde/ketone and ester	N-H bond

**Table 2a:** ANOVA for a mixture of cubic model.

Source	Sum of squares		df	Mean square		F value	Prob > F
	D	AV		D	AV		
Model	1233.92	7.60	9	137.10	0.84	6.366 × 10^−7^	< 0.0001
LM	988.87	5.96	2	494.44	2.98	6.366 × 10^−7^	< 0.0001
X_1_X_2_	8.57	4.085 × 10^−3^	1	8.57	4.085 × 10^−3^	6.366 × 10^−7^	< 0.0001
X_1_X_3_	107.68	0.01	1	107.68	0.01	6.366 × 10^−7^	< 0.0001
X_2_X_3_	5.02	0.65	1	5.02	0.65	6.366 × 10^−7^	< 0.0001
X_1_X_2_X_3_	3.90	6.045 × 10^−3^	1	3.90	6.045 × 10^−3^	6.366 × 10^−7^	< 0.0001
X_1_X_2_(X_1_-X_2_)	28.80	0.29	1	28.80	0.29	6.366 × 10^−7^	< 0.0001
X_1_X_3_(X_1_-X_3_)	0.77	0.43	1	0.77	0.43	6.366 × 10^−7^	< 0.0001
X_2_X_3_(X_2_-X_3_)	90.31	0.04	1	90.31	0.04	6.366 × 10^−7^	< 0.0001

LM = linear mixture, D = density, AV = acid value

**Table 2b:** Point prediction.

Name	Prediction	SE Mean	95% CI low	95% CI high	SE pred.	95% PI low	1.81
Acid value	1.81	0.00	1.81	1.81	0.00	1.81	1.81
Density	907.13	0.00	907.13	907.13	0.00	907.13	907.13
Component	Name	Level	Low level	High level	Std. Dev		

X_1_	*CPO*	0.33	0.00	1.00	0.00		
X_2_	CAO	0.33	0.00	1.00	0.00		
X_3_	PO	0.34	0.00	1.00	0.00		

Final equations in term of real component:

**Table 3a:** Experimental design for FAME synthesized.

Variables	Units	Symbol	Levels				
			-2	-1	0	1	2
Reaction time	(min)	*P*_2_	50	55	60	65	70
MCCP amount	(wt.%)	*P*_2_	3.0	3.5	4.0	4.5	5.0
Reaction temp.	(°C)	*P*_3_	60	65	70	75	80
MeOH/OMR	(ml/ml)	*P*_4_	3	4	5	6	7

MeOH/OMR = Methanol/oil molar ratio

**Table 3b:** Pro-catalytic activity of catalysts calcined at 700 °C for 4 h

Catalysts	S_BET_ (m^2^g^−1^)	Total pore volume (cm^3^g^−1^)	%CaO	BS (μmole.g^−1^)			TBS	BSD (μmole.m^−2^)
				Weak < 450 °C	Medium >450 °C	Strong >650 ^o^C		
*CCL*	0.80	5 × 10^−3^	74.60	-	30	116	146	182.50
*CMA*	0.80	5 × 10^−3^	62.80	-	22	102	124	155.00
*MCCP*	1.00	5 × 10^−3^	78.74	8	32	143	183	183.00

BS = Basic site, TBS = Total basic site, BSD = Basic site density = TBS/N_2_-AA, CCL = Calcined Citrullus lanatus, CMA = Calcined Musa acuminate, MCCP = Mixed calcined catalyst powder

**Table 4a:** FAME result of experimental run, predicted and the residual value

Std	Run	Block	P_1_	P_2_	P_3_	P_4_	FAME (wt. %)	Predicted FAME (wt. %)	Residual
1	12	1	-1.000	-1.000	-1.000	-1.000	83.90	83.99	-0.094
2	3	1	1.000	-1.000	-1.000	-1.000	83.90	84.01	-0.11
3	6	1	-1.000	1.000	-1.000	-1.000	88.24	88.34	-0.10
4	30	1	1.000	1.000	-1.000	-1.000	87.90	87.95	-0.049
5	28	1	-1.000	-1.000	1.000	-1.000	87.74	87.75	-0.013
6	2	1	1.000	-1.000	1.000	-1.000	89.27	89.37	-0.095
7	29	1	-1.000	1.000	1.000	-1.000	91.09	91.19	-0.095
8	4	1	1.000	1.000	1.000	-1.000	92.29	92.39	-0.10
9	22	1	-1.000	-1.000	-1.000	1.000	84.42	84.20	0.22
10	19	1	1.000	-1.000	-1.000	1.000	88.21	87.93	0.28
11	11	1	-1.000	1.000	-1.000	1.000	90.92	90.64	0.28
12	20	1	1.000	1.000	-1.000	1.000	94.10	93.97	0.13
13	23	1	-1.000	-1.000	1.000	1.000	83.79	83.56	0.23
14	1	1	1.000	-1.000	1.000	1.000	89.11	88.89	0.22
15	8	1	-1.000	1.000	1.000	1.000	89.31	89.08	0.23
**16**	**5**	**1**	**1.000**	**1.000**	**1.000**	**1.000**	**94.29**	**94.01**	**0.28**
17	7	1	-2.000	0.000	0.000	0.000	84.50	84.68	-0.18
18	10	1	2.000	0.000	0.000	0.000	89.49	89.62	-0.13
19	27	1	0.000	-2.000	0.000	0.000	80.34	80.51	-0.17
20	14	1	0.000	2.000	0.000	0.000	89.85	89.98	-0.13
21	15	1	0.000	0.000	-2.000	0.000	89.92	90.05	-0.13
22	26	1	0.000	0.000	2.000	0.000	93.68	93.85	-0.17
23	18	1	0.000	0.000	0.000	-2.000	89.54	89.06	0.48
24	13	1	0.000	0.000	0.000	2.000	90.10	90.88	-0.78
25	25	1	0.000	0.000	0.000	0.000	90.20	90.13	0.075
26	21	1	0.000	0.000	0.000	0.000	90.10	90.13	-0.025
27	24	1	0.000	0.000	0.000	0.000	90.12	90.13	-0.005
28	17	1	0.000	0.000	0.000	0.000	90.11	90.13	-0.015
29	16	1	0.000	0.000	0.000	0.000	90.12	90.13	-0.094
30	9	1	0.000	0.000	0.000	0.000	90.10	90.13	-0.025

**Table 4b:** Test of significance for every regression coefficient

Source	Sum of squares	df	Mean Square	F-value	P-value
Model	303.37	14	21.67	215.57	< 0.0001
P_1_	36.61	1	36.61	364.15	< 0.0001
P_2_	134.52	1	134.52	1338.23	< 0.0001
P_3_	21.70	1	21.70	215.85	< 0.0001
P_4_	4.99	1	4.99	49.61	< 0.0001
$P_{1}^{2}$	15.21	1	15.21	151.28	< 0.0001
$P_{2}^{2}$	40.80	1	40.80	405.85	< 0.0001
$P_{3}^{2}$	5.72	1	5.72	56.90	< 0.0001
$P_{4}^{2}$	0.040	1	0.040	0.40	0.5361
P_1_*P*_2_	0.16	1	0.16	1.63	0.2209
P_1_P_3_	2.56	1	2.56	25.47	0.0001
P_1_P_4_	13.84	1	13.84	137.67	< 0.0001
P_2_P_3_	0.84	1	0.84	8.33	0.0113
P_2_P_4_	4.39	1	4.39	43.66	< 0.0001
P_3_P_4_	19.36	1	19.36	192.60	< 0.0001
Residual	1.51	15	0.10	-	-
Lack of Fit	1.50	10	0.15	104.94	0.3072
Pure Error	0.00715	5	0.0014	-	-
Cor Total	304.88	29			

**Fits statistics**					
R squared	99.51%				
Adjusted R squared	99.04%				
Predicted R squared	97.16%				
Adequate precision	60.219

**Table 5:** Properties of TMO and FAME.

Parameter	TMO	FAME	ASTM D6751	EN 14214 [1]
Density (kg/m^3^) @ 25 ^o^C	907	886	-	860-900
Viscosity @ 40 ^o^C/ (mm^2^/s)	4.40	2.10	1.9-6.0	3.5-5.0
Moisture content (%)	0.002	0.001	<0.03	0.02
%FFA (as oleic acid)	0.90	0.40	0.40 max	0.25 max
Acid value (mg KOH/g oil)	1.80	0.20	0.80 max	0.5 max
Iodine value (g I_2_/100g oil)	98.20	80.52	-	120 max
Saponification value (mg KOH/g oil)	172.00	140.20	-	-
Peroxide value (meq O_2_/kg oil)	10.20	11.21	-	12.85
HHV (MJ/kg)	40.91	42.47	-	-
Cetane number	55.93	67.10	57 min	51 min
API	24.51	28.21	39.95 max	-
Diesel index	63.76	79.31	50.4 min	-

TMO = Ternary mixed oil

**Final equation in term of coded**

(3) $$\begin{array}{cc} \text{FAME}= & \;+90.13+1.23X_{1}+2.37X_{2}+0.95X_{3}+0.46X_{4}-0.10X_{1}X_{2}+0.40X_{1}X_{3\;}+0.93X_{1}X_{4}\\ & \;-0.23X_{2}X_{3}+0.52X_{2}X_{4}-1.10X_{2}X_{3}-0.74X_{1}^{2}-1.22X_{2}^{2}+0.46X_{3}^{2}-0.038X_{4}^{2} \end{array}$$

## 2 Experimental Design, Materials, and Methods

Response surface methodology (Simplex lattice design) and central composite design (expert 6.0.8 trial version) were employed to determine the blend ratio and the effects of variation of reaction time, catalyst amount, reaction temperature and methanol to oil molar ratio on the FAME synthesized. Materials used include CH_3_OH, Ethanol, Sulphuric acid, Wij's solution, etc. (ChemiSciences Nig. Ltd.), Cucurbita pepo-Chrysophyllum albidum-papaya seeds, Citrullus lanatus and Musa acuminate peels. Equipment used are scanning electron microscopy (SEM) to examine the surface morphology of the calcined catalysts (CaO) derived from the mixture of Citrullus lanatus and Musa acuminate peels calcined powder, energy dispersive spectroscope for determination of elemental analysis of the samples and the quantitative composition of the catalysts, X-ray diffraction analysis equipped with Kά and Cu radiation source, accelerated at 20 mA and 40 kV used to determine the angular scanning electron performed in the range of 20^o^ <2θ <80^o^ at speed of 2 °C min-1, Fourier transform infrared spectroscopy used for determination of the presence of functional group and verify the presence of characteristic absorption bands of CaO, and QUANTACHROME, 1 KE, BET isothermal sorption was used to determination of the surface area of the catalysts through N_2_-adsorption CO_2_ TPD thermal.

Cucurbita pepo-Chrysophyllum albidum-papaya seeds were washed with deionized water to remove dirt's, sun dried for 15 days until a constant weight was achieved before milled to powders.

The solvent extraction method by the Soxhlet apparatus was used for oil extraction from the powders. 100 g each powder was measured, tightly placed in a muslin bag, and the solvent, n-hexane was measured into the round bottom flask of Soxhlet extractor. A 4-place combo heating mantle unit was loaded with four 500 ml capacity Soxhlet extractors. The reaction time was 60 min and the heating temperature was adjusted to the temperature range of 68-70 °C. At the end of the reaction, excess n-hexane in the extracted oil was recycled using an evaporator. The percentage of oil-free of n-hexane was determined using the ratio of the Eq. (4)

(4) $$Oil\;yield\;\%\;\left(v/v\right)=\frac{W_{OIL}}{\;W_{POWDER}}\;X\;100\;$$

Ternary oil blend was carried out by using three variables (Cucurbita pepo oil, Chrysophyllum albidum oil, and papaya oil) as input factors and two response variables (density and acid value). The simplex lattice design predicted a ratio of 33:33:34 ternary blend, this was used for oil mixed and the oil was kept in the jar.

Citrullus lanatus and Musa acuminate peels were washed to remove dirt, then oven-dried to constant weight in an electrical oven. The dried peels were milled into powders, separated into smaller particle sizes using a mesh strainer (mesh size: 125 mm-20 μm) to aid calcination. Each of the powder and the blend (100 g Citrullus lanatus peel powder + 100 g Musa acuminate peel powder) were calcined at 700 °C for 4 h in an electrical furnace. After cooling, the calcined powders were characterized using scanning electron microscopy, energy dispersive spectroscope, X-ray diffraction analysis equipped with Kά and Cu radiation source, accelerated at 20 mA and 30 kV, with angular scanning electron performed in the range of 20^o^ <2θ <80^o^ at speed of 2 °C min-1, Fourier transform infrared spectroscopy, and BET isothermal adsorption and Hammett indicator method [12].

For FAME synthesized, the predicted acid value of 1.81 mg KOH/g oil was validated as 1.80 mg KOH/g oil (FFA = 0.90) through the design, the ternary mixture of the oil (TMO) containing 33:33:34 of PO: CAO: PO blend meets the require conditions for biodiesel production via transesterification with catalyzed methanolysis of derived based catalyst CaO (d-CaO). FAME was synthesized through the procedure employed by [12] with little modifications on data factor varied and catalyst reusability steps as follows: A three-necked-reactor was used to carry out the FAME production, a total of thirty experimental runs was generated and carried out via four variable factors were considered namely; reaction time of 50-70 min, MCCP amount 3.0-5.0 (wt.), reaction temperature of 60-80 °C, and MeOH/OMR of 3-7, respectively. Initially, 80 ml of the oil was preheated at 60 °C for 1 h, a measured catalyst amount was added to a measured volume of methanol in 250 ml flask, heated at 65 °C for 20 min, and then transferred into the preheated oil in the reactor, and the reaction was monitored for a period of time until it reaches completion. At the end of the reaction, the catalyst was separated by decantation and the biodiesel phase was separated from the methanol phase by separating funnel. The leach catalyst in the biodiesel was removed by washing with a mixture of 2.0 g NaCO_3_ and 40 ml ethanol thermally heated for 2 h under agitation. The mixture was filtered, washed with distilled water trice before the separation of biodiesel through gravity settling was carried out. Washed biodiesel was then dried over anhydrous Na_2_SO_4_, and then separated by filtration to obtain pure biodiesel (FAME).

For catalyst reusability, the derived CaO was recycled for reuse at the end of the reaction with reduction in the 4th, 5th and 6th cycle. Hence, the catalyst reusability was stopped after 3rd usage. For experimental design for FAME synthesized, a central composite design was used to generate a total of 30 (thirty) experimental runs, which includes the plus and minus axial points, plus and minus factorial points and the central-point with factors low and high entered in terms of alpha. For every combination of categorical factor levels, central composite design was duplicated.

The density, viscosity, the moisture content, acid value, the iodine value, and the peroxide value of the mixed oil were higher than the FAME values confirming that the synthesized product is consistent with biodiesel and that the conversion of mixed oil to FAME was complete with negligible resistance to flow and reduce internal drag in engine.

The ternary mixed ratio of oil is shown in Table 1b and Fig. 1. The SEM image and FTIR of the calcined catalysts and the mixed catalyst are shown in Fig. 2(a-b). Compositions of the calcined catalyst by XRD and BET sorption are listed in Table 3. Variable factors, the experimental yield, and the predicted value data for FAME are illustrated in Table 4a, while the test of significant and fits statistics by CCD optimization are shown in Table 4b. Fig 4(a-b), displayed the predicted against the actual FAME yield, as well as the three-dimensional plots that exist between the four factors and the FAME response. Table 5 provides the properties of the ternary mixed oil (TMO) and the FAME produced.

## Appendix Supplementary materials

Image, application 1
